# The Association of Food Groups and Consumption Time with Hyperuricemia: The U.S. National Health and Nutrition Examination Survey, 2005–2018

**DOI:** 10.3390/nu15143109

**Published:** 2023-07-12

**Authors:** Yuanyuan Wang, Ruiming Yang, Ziteng Cao, Sijia Han, Tianshu Han, Wenbo Jiang, Xinyang Wang, Wei Wei

**Affiliations:** 1 Department of Nutrition and Food Hygiene, The National Key Discipline, School of Public Health, Harbin Medical University, Harbin 150081, China; 2 Department of Pharmacology, College of Pharmacy Key Laboratory of Cardiovascular Research, Ministry of Education, Harbin Medical University, Harbin 150081, China

**Keywords:** hyperuricemia (HUA), food consumption time, national health and nutrition examination survey (NHANES), food patterns equivalents database (FPED), food groups

## Abstract

Hyperuricemia (HUA) is associated with a wide range of diseases and increases the public health burden on society as a whole. In addition to genetic variation, diet plays a crucial role in the prevention and treatment of HUA as an important modifiable behavior. The purpose of this study is to investigate whether food groups and consumption time are associated with HUA. A total of 41,230 participants from the National Health and Nutrition Examination Survey between 2005 and 2018 were included in the study. All meals, including breakfast, lunch, and dinner, were obtained according to their corresponding Food Patterns Equivalents Database dietary data. The binary logistic regression model was used to analyze the relationship between food groups, food consumption time and HUA. We found that the intake of fruit (mixed in various forms) (OR = 0.942, 95% CI: 0.909–0.976) or freshly squeezed juices (OR = 0.915, 95% CI: 0.859–0.975), milk (OR = 0.839, 95% CI: 0.808–0.872), and eggs (OR = 0.881, 95% CI: 0.839–0.924), poultry (OR = 1.055, 95% CI: 1.033–1.077) and seafood high in *n*-3 fatty acids (OR = 1.068, 95% CI: 0.1.018–1.120) at dinner, eating refined grains at breakfast (OR = 0.954, 95% CI: 0.924–0.985) and dinner (OR = 0.962, 95% CI: 0.944–0.980), eating whole grains (OR = 0.908, 95% CI: 0.845–0.976) at lunch, consuming alcoholic beverages or foods at breakfast (OR = 0.748, 95% CI: 0.564–0.990)/lunch (OR = 1.118, 95% CI: 1.008–1.240)/dinner (OR = 1.127, 95% CI: 1.073–1.185) were associated with HUA. Eating particular meals at particular times of the day was related to a lower risk of HUA.

## 1. Introduction

Hyperuricemia (HUA) is a chronic metabolic disease caused by the accumulation of serum uric acid (UA) due to purine metabolism disorders [1]. Studies have shown that the prevalence of HUA more than doubled between the 1960s and the 1990s and continued to increase steadily afterwards [2,3,4]. Regional differences in the prevalence of HUA due to the diversity of dietary patterns, socioeconomic conditions and genetics, with a higher prevalence in developed countries than in developing countries [5]. In recent years, the prevalence of HUA among adults in the United States, Australia and South Korea is 20.1%, 16.6% and 11.4% [2,6], respectively. Meanwhile, the prevalence of HUA is 10.6% in Thailand and 8.4% in Saudi Arabia [7]. HUA is associated with diabetes, chronic kidney disease, cardiovascular disease and other diseases, which greatly increases the public health burden on society as a whole [1,8].

Recent studies have revealed that diet, in addition to genetic variants, is an essential modifiable behavior that plays a critical role in the prevention and treatment of HUA, even though the risk factors for HUA have not yet been fully identified [9]. Traditional low-purine dietary approaches used to treat HUA have limited efficacy and sustainability; therefore, there is an urgent need for effective dietary strategy to address the morbidity burden of HUA. Most of the previous diet-related research has concentrated on the impact of particular nutrients on HUA. For example, in animal models, a high-protein diet induces the development of HUA [10], whereas a low-fat and high-vitamin diet may help prevent the development of HUA [11]. There is little research on the relationship between food groups and HUA. Meanwhile, in recent years, overwhelming animal studies have demonstrated that ingestion time is another major factor for the well-being of organisms because of the circadian effects [12]. The timing of food intake may also be an important entry point for influencing HUA. However, very few research have looked at the association of consumption time of foods with HUA.

With the development of economy and the change in lifestyle, people’s dietary groups have become diversified. In order to further understand the impact of diet on HUA, we combined the 2005–2018 National Health and Nutrition Examination Survey (NHANES) and Food Patterns Equivalents Database (FPED) to evaluate the influence of dozens of food groups and food consumption time on HUA. Since not everyone has access to nutritionists, the study’s findings can help people choose the appropriate diet.

## 2. Materials and Methods

### 2.1. Data Source and Study Population

The NHANES is a representative multistage stratified sampling health survey in the United States. Details of the sampling methods and data collection have been provided elsewhere [13]. FPED is used to evaluate whether the food and beverage intake of Americans meets the recommendations of the dietary guidelines for Americans.

**Figure 1:**
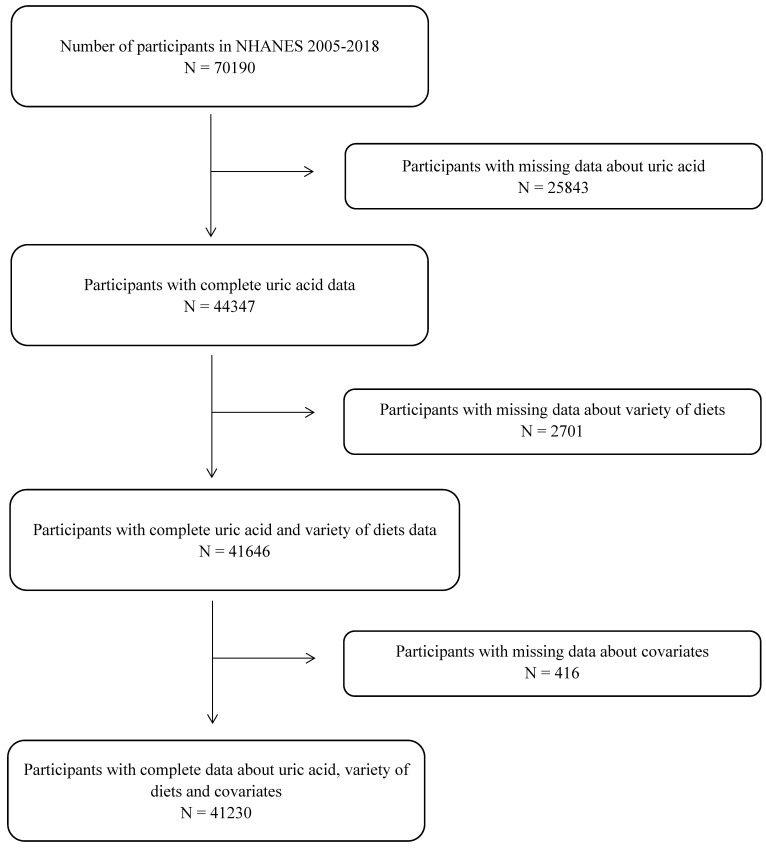
Flowchart for the selection of eligible participants.

Data for older adults 18 years or more from the 2005–2018 NHANES and the corresponding FPED version 2005–2018 were used for this study. Excluding those lacking serum UA information and those lacking study factors and covariates, ultimately a total of 41,230 participants were included (Figure 1). Institutional review board approval of the National Center for Health Statistics and written informed consent were obtained prior to data collection.

### 2.2. Dietary Assessment

The Federal Government, as part of its ongoing nutrition monitoring and surveillance activities conducts the “What We Eat in America” (WWEIA) survey, which is the component of the NHANES 2005–2018 collected dietary information by using an interviewer-administered 24 h recall that used automated multiple pass methodology developed by the U.S. Department of Agriculture (USDA) [14]. A second dietary recall, 3–10 days after the first dietary recall, was obtained by using phone calls. Although two 24 h dietary recalls were collected in the 2007–2010 NHANES, only the first recall data are recommended to be used by the NCHS as different methods were used to collect dietary data, i.e., day 1 by in-person and day 2 by phone calls [15]. A single 24 h recall has been reported to be adequate to estimate mean group dietary intake [16]. To assess the intake of major food groups, the same definitions were used for the same food groups in the FPED and MyPyramid Equivalents Database across different survey cycles [13].

### 2.3. Main Exposure

The FPED converts foods to the respective number of cup equivalents of fruit, vegetables, and dairy; ounce equivalents of grains and protein foods; number of alcoholic drinks; teaspoon equivalents of added sugars; and gram equivalents of solid fats and oils. The main components of food groups are further subdivided to facilitate in-depth analysis of the data. For example, the red and orange vegetables are subdivided into tomatoes and other red and orange vegetables. Detailed breakdowns were shown in Supplementary Table S1. The variable “Name of eating occasion” divided the food groups into breakfast, lunch and dinner according to the time of intake of the food group. The next step in the analysis was performed for those who consumed food at breakfast/lunch/dinner.

### 2.4. Outcome Variable and Covariates

The outcome variable was HUA status, which was determined by the serum UA level. HUA was defined as serum UA levels ≥ 7 mg/dL for males and ≥6 mg/dL in females [17].

The following covariates were included in the analyses: age (years), gender (males/females), race/ethnicity (non-Hispanic white, non-Hispanic black, Hispanic (Mexican-American and other Hispanic), and other race/multiracial), annual household income (over USD 75,000/less than or equal to USD 7500) and education (less than high school, high school graduate, college or more). Drinking condition was assessed by the following question, “Had you had drinks for at least 12 times in the last 12 months?”. Responses include “yes” and “no”. The exercise was assessed by using the question, “Have you participated in exercise in the past month?”. Responses include “yes” and “no”. Smoking-related variable (Do you smoke cigarettes now?). Responses include “yes” and “no”. Body mass index (BMI) was calculated by dividing measured weight (kg) by measured height (m^2^). Participants were classified as having diabetes mellitus (DM) based on: the “yes” answer to the questions: “Have you ever been told by a doctor or health professional that you have diabetes or sugar diabetes?” or “now taking insulin” or “now taking diabetic pills” or hemoglobin A1c was greater than or equal to 6.5%, or fasting (8–24 h) plasma glucose was greater than or equal to 7.0 mmol/L, or random plasma glucose/two-hour OGTT plasma glucose greater than or equal to 11.1 mmol/L. Participants were classified as pre-DM based on the “yes” answer to the questions: “Have you ever been told by a doctor or health professional that you have prediabetes?” or hemoglobin A1c was 5.7–6.5 (%) or fasting (8–24 h) plasma glucose was 5.6–7.0 (mmol/L) or their random plasma glucose/two-hour OGTT plasma glucose was 7.8–11.0 (mmol/L). Participants were classified as having hypertension if their systolic blood pressure was greater than or equal to 140 mmHg or diastolic blood pressure was greater than or equal to 90 mmHg, or they were currently taking medication to lower high blood pressure. Hyperlipidemia was defined as the presence of one or more of the following serum measures: total cholesterol > 200 mg/dL; triglycerides > 200; high-density lipoproteins < 40 mg/dL; low-density lipoproteins > 130 mg/dL. Current use of cholesterol-lowering medications classified an individual as hyperlipidemia. Estimated glomerular filtration rate (eGFR) was calculated using the CKD-EPI creatinine equation, as previously described [18]. Chronic Kidney Disease (CKD) was defined as eGFR < 60 mL/min/1.73 m^2^ or albuminuria, which was defined as an albumin-to-creatinine ratio (ACR) above 30 mg/g [19].

### 2.5. Statistical Analysis

Sample weights, clustering, and stratification were incorporated in all analyses because of the complex sampling design of the NHANES, as required to analyze the NHANES data. Demographic characteristics, dietary nutrient intake, and anthropometric measurements were presented as mean ± standard deviation for continuous variables and numbers (% percentage) for categorical variables. Continuous variables were analyzed by Student’s *t*-tests and categorical variables were analyzed with the chi-square tests. Binary logistic regression models were used to estimate the β coefficients and 95% confidence intervals (CI) for the associations between food group and HUA.

All data were analyzed using R (version 3.5.3), As there were relatively numerous food group variables, the *p*-values of the two independent sample *t*-tests were corrected using FDR to reduce false positives. *p*-value/adjusted *p*-value were two-tailed (α = 0.05). *p*-value/adjusted *p*-value less than 0.05 were considered significant.

### 2.6. Sensitivity Analysis

We performed 5 kinds of sensitivity analyses. In the first sensitivity analysis, we conducted a subgroup analysis based on gender to check whether gender could influence the relationship between food groups and HUA. In the second sensitivity analysis, we grouped according to hypertension status to examine whether hypertension status could influence the relationship between food groups and HUA. In the third sensitivity analysis, we grouped the food groups according to CKD status to check whether CKD status could influence the relationship between food group and HUA. In the fourth sensitivity analysis, we grouped the groups according to drinking status to check whether drinking status could influence the relationship between food groups and HUA. In the fifth analysis, we excluded the HUA patients, with a follow-up duration <2 years to examine the impact of severe illness or accident on the results.

## 3. Results

### 3.1. Characteristics of the Study Population

Characteristics of the study population from the seven cycles of NHANES (2005–2018) surveys were shown in Table 1. A total of 41,230 people joined our study through screening; based on the weight, it corresponded to 241,876,384 people in the United States. Compared with the non-HUA population, patients with HUA were more likely to be male, non-Hispanic white, had a lower household income, level of exercise and smoking rate, had higher age, education level, marriage rate, drinking rate, BMI and prevalence of DM, pre-DM, hypertension, hyperlipidemia and CKD.

### 3.2. Characteristics of the Food Group

FPED categorized the diets of people in the corresponding NHANES into dozens of food groups according to their composition. A two-group independent samples Student’s *t*-tests was performed to understand the relationship between food groups and HUA, corrected for p-values due to the large number of variables. All the corresponding food groups of the study population were shown in Table 2. Compared with non-HUA population, patients with HUA ate less fruit (whether whole fruit or juicing), grain, dairy products and sugar, but more meat and protein-rich foods (except legumes), white potatoes, seafood high in *n*-3 fatty acids, and alcohol.

### 3.3. Association between Food Groups and HUA

**Figure 2:**
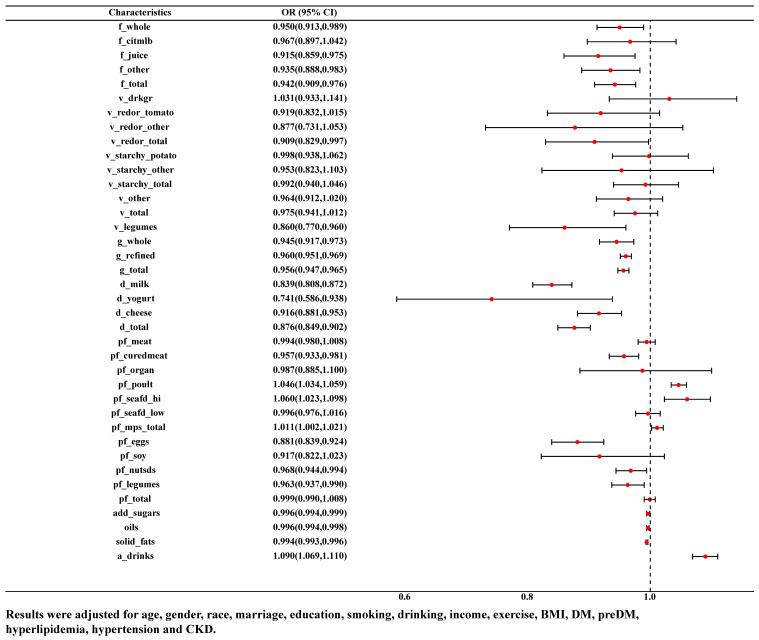
ORs and 95% CI for the association of variety of food groups with HUA.

The association between food groups and HUA was examined by binary logistic model. As was shown in Figure 2, after adjusting for age, gender, race, marriage, education, smoking, drinking, income, exercise, BMI, DM, pre-DM, hyperlipidemia, hypertension and CKD, the consumption of citrus, melon and berry fruits was negatively correlated with HUA (OR = 0.942, 95% CI: 0.909–0.976), whether in whole fruit (OR = 0.950, 95% CI: 0.913–0.989), juice (OR = 0.915, 95% CI: 0.859–0.975) or other forms (OR = 0.935, 95% CI: 0.888–0.983). Consumption of red and orange vegetables (OR = 0.909, 95% CI: 0.829–0.997) as well as legume vegetable (OR = 0.860, 95% CI: 0.770–0.960) and grains (either whole grains (OR = 0.945, 95% CI: 0.917–0.973) or refined grains (OR = 0.960, 95% CI: 0.951–0.969)) were negatively correlated with HUA. Consumption of more dairy products (whether milk (OR = 0.839, 95% CI: 0.808–0.872), yoghurt (OR = 0.741, 95% CI: 0.586–0.938), cheese (OR = 0.916, 95% CI: 0.881–0.953) or combination (OR = 0.876, 95% CI: 0.849–0.902)) and other protein-rich foods (cured meats (OR = 0.957, 95% CI: 0.933–0.981), eggs (OR = 0.881, 95% CI: 0.839–0.924), beans and peas (OR = 0.963, 95% CI: 0.937–0.990), nuts and seeds (OR = 0.968, 95% CI: 0.944–0.994)) was negatively correlated with HUA. However, more poultry meat (OR = 1.046, 95% CI: 1.034–1.059), seafood rich in *n*-3 fatty acids (OR = 1.060, 95% CI: 1.023–1.098), alcohol (OR = 1.090, 95% CI: 1.069–1.110) were positively correlated with HUA.

### 3.4. Association between Food Intake Time and HUA

**Figure 3:**
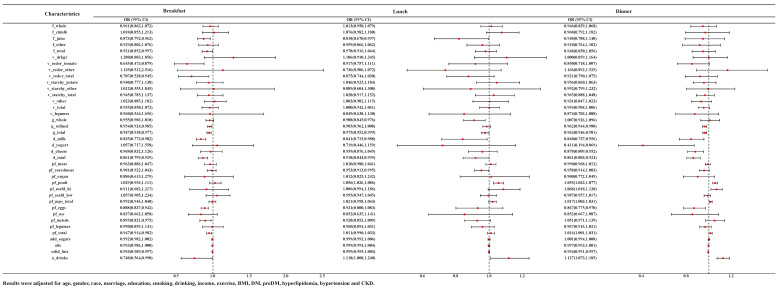
ORs and 95% CI for the association of variety of food groups consumption time with HUA.

In NHANES, staff have divided the food into multiple time periods of intake based on the timing of the food intake. We selected seven NHANES cycles in which people with food intake at breakfast/lunch/dinner were individually subjected to logistic regression to understand the relationship between food groups and HUA at different eating times. Saw the Supplementary Tables S2–S4 for the baseline data for people corresponding to each time period. The association between food groups and HUA at different eating times was displayed in Figure 3. After adjusting for age, gender, race, marriage, education, smoking, drinking, income, exercise, BMI, DM, pre-DM, hyperlipidemia, hypertension and CKD, we found that juice (OR = 0.873, 95% CI: 0.792–0.962), milk (OR = 0.835, 95% CI: 0.773–0.902), tomatoes (OR = 0.645, 95% CI: 0.473–0.879) and red orange vegetables (OR = 0.707, 95% CI: 0.528–0.945), refined grains (OR = 0.954, 95% CI: 0.924–0.985) and eggs (OR = 0.888, 95% CI: 0.837–0.942) at breakfast, whole grains (OR = 0.908, 95% CI: 0.845–0.976), juice (OR = 0.818, 95% CI: 0.670–0.997) and cured meats (OR = 0.953, 95% CI: 0.913–0.995) at lunch, refined grains (OR = 0.962, 95% CI: 0.944–0.980), eggs (OR = 0.867, 95% CI: 0.775–0.970) and dairy products of any kind (milk (OR = 0.840, 95% CI: 0.737–0.956), yogurt (OR = 0.411, 95% CI: 0.194–0.869), and cheeses (OR = 0.878, 95% CI: 0.809–0.952)) at dinner were negatively correlated with HUA. At the same time, eating poultry at lunch (OR = 1.056, 95% CI: 1.026–1.086) or dinner (OR = 1.055, 95% CI: 1.033–1.077) and seafood rich in *n*-3 fatty acids (OR = 1.068, 95% CI: 1.018–1.120) at dinner were deemed to be positively correlated with HUA. Interestingly, the consumption of alcohol at breakfast (OR = 0.748, 95% CI: 0.564–0.990) was negatively correlated with HUA, but positively correlated with HUA at lunch (OR = 1.118, 95% CI: 1.008–1.240) and dinner (OR = 1.127, 95% CI: 1.073–1.185).

### 3.5. Sensitivity Analysis

In the first sensitivity analysis (Supplementary Table S5), after adjusting for gender, the effects were relatively stable for most food groups. On the basis of the results of Figure 1, most food groups remained associated with the occurrence of HUA after excluding gender. Eating fruits (whether or not citrus, melon and berry fruits), cheeses, refined grains and eggs and drinking milk were thought to be negatively correlated with HUA. However, the consumption of poultry meat and alcohol could be positively correlated with HUA.

In the second sensitivity analysis (Supplementary Table S6), excluding the effect of hypertension, among the food groups that continue to be associated with the development of HUA were fruit juices, refined grains, milk, poultry, eggs and alcohol.

In the third sensitivity analysis (Supplementary Table S7), excluding the effect of CKD, whole and refined grains, milk and eggs continue to be negatively correlated with HUA, while seafood high in *n*-3 fatty acids and alcohol were positively correlated with HUA.

In the fourth and the final sensitivity analysis (Supplementary Tables S8 and S9), people were classified according to whether they drank or not; participants who did had a follow-up duration of <2 years. Overall, fruits, grains, milk, poultry, seafood rich in *n*-3 fatty acids, eggs and alcohol continued to be associated with HUA.

## 4. Discussion

In this study, we discovered a relationship between various food groups and the prevalence of HUA using population data from NHANES (2005–2018) and related food group data from FPED. At the same time, we used data from the timing of food category intake in the NHANES, which was broken down into breakfast, lunch, and dinner, to discover that eating particular meals at particular times of the day was related to a lower risk of HUA.

FPED is the new name for the former MyPyramid Equivalents Database (MPED) developed by the United States Department of Agriculture, Agricultural Research Service (ARS), Food Surveys Research Group (FSRG). Single-ingredient foods (such as orange juice, jacket potatoes, rice, grilled fish or skimmed milk) can be assigned directly to fruit, vegetables, grains, protein foods and dairy components, respectively. However, many foods such as pizzas, sandwiches, fruit salads, chocolate shakes, fried eggs and casseroles are multi-ingredient foods. Direct analysis would be complex and would also increase the workload, making the results difficult to interpret. Therefore, it is necessary to break down multi-component foods into multiple food groups before analysis can be carried out. It is extremely exciting that FPED has achieved this.

To our knowledge, this was the first and relatively large population study based on NHANES, which used a nationally representative sample of U.S. adults to reveal an association between dozens of food groups in daily life with HUA. Our results found that increased consumption of fruits, grains, dairy products and eggs was negatively correlated with HUA. However, a higher consumption of poultry meat, seafood high in *n*-3 fatty acids and alcohol might be positively correlated with HUA. We found that the protective effect of fruit intake in the form of 100% fruit juice was relatively stable for HUA. This may be because the nutrients in fresh fruit juice are similar to those in whole fruit. In the process of making fruit juice, the blender cuts the fiber of the fruit into smaller pieces for better absorption. Although fruit juice contains fructose, juicing breaks down the cell walls within the fruit, resulting in the release of more polyphenols [20]. Study has shown that polyphenols in 100% fruit juice may inhibit the absorption of natural sugars [21]. At the same time, sensitivity analysis showed that fruit consumption is not protective against HUA in CKD patients, which may be caused by the inability of the kidney to properly excrete UA produced by fructose metabolism in fruit [22]. As the body’s primary organ for excreting uric acid, the kidneys account for excreting two-thirds of the body’s UA salts. Clinicians frequently struggle to control HUA when renal illness is present, indicating that renal function can have an impact on HUA [23]. This may possibly be the cause of the greater prevalence of CKD patients among HUA patients as indicated by our findings.

There is evidence that whole grains lower the risk of HUA [24]. Despite current dietary recommendations favoring whole grains over refined grains [25], it is challenging to completely replace refined grains with whole grains. There is nothing wrong with eating refined grains, but it is important to balance your intake of whole grains with refined grains. According to our research, consuming whole grains for lunch and refined grains for breakfast may be negatively correlated with HUA. A traditional wheat breakfast is associated with a reduced risk of HUA [26]. However, switching the intake of whole grains from morning to lunch is associated with a reduced risk of cardiovascular disease and all-cause mortality [12]. Unfortunately, there are not many studies on the connection between grains and HUA. Except for one study that demonstrated a 0.90% reduction in the incidence of HUA for every 10g increase in refined grain intake [27] there are not many studies that clearly demonstrate an independent association between refined grains and HUA. More research is required to determine whether the correlation between refined grains and HUA discovered in our study was directly attributable to a decrease in the consumption of purine-rich foods brought on by eating more refined grains.

The protective effects of milk and eggs against HUA are mostly unchanged. As far as we know, milk consumption can increase UA excretion and lower serum UA levels [28]. Meanwhile, people who exhibit sensitivity to milk allergens are more likely to develop HUA than people who do not [29]. Eating eggs for breakfast helps to maintain post-meal blood glucose levels as well as daily nutrition because eggs are a fantastic source of high-quality protein and contain a number of important nutrients and antioxidants [30,31]. We found that eating eggs for dinner was negatively related to HUA; it is possible that the consumption of eggs increases satiety, which reduces food intake, and studies have demonstrated the potential benefit of reducing energy intake at dinner. In addition, our results indicated that poultry meat, seafood high in *n*-3 fatty acids and alcoholic beverages and foods, which have been shown to be linked to higher UA [32,33,34]. It is generally known that UA is the end product of purine metabolism and that consuming foods high in purines is linked to the production of HUA. Both poultry meat and seafood rich in *n*-3 fatty acids are high in purines. In a study in China, HUA was found to be associated with greater consumption of meat and *n*-3 fatty acid-rich seafood [35], and this was also supported by a study conducted in the USA [27]. Our research indicates that poultry meat and seafood high in *n*-3 fatty acids were positively related with HUA when consumed at lunch and dinner. A study examined the circadian rhythm of UA in adult males found that UA changes in gradually increasing concentrations throughout the night and peaking in the morning, which may conceal the effect of poultry meat and seafood on HUA at breakfast.

In our study, we also found that the prevalence of HUA was significantly higher in men than in women, which was consistent with previously reported findings that in recent years, the prevalence of HUA in Chinese men reached 21.6% compared to 8.6% in women [36]. This may be due to the ability of estrogen to promote uric acid excretion [37]. According to previous reports, US and European guidelines for the management of HUA recommend restricting the intake of animal-derived foods, but not purine-rich vegetables or purine-rich fruits [27]. In our study, the consumption of vegetables had no effect on HUA, whether they were high- or low-purine vegetables. The Dietary Approaches to Control Hypertension (DASH) diet can also reduce serum UA levels, which emphasizes the intake of fruits, vegetables, and dairy products and reduces saturated fat, is consistent with our results [38]. The desire to include a small amount of alcohol in your regular diet or to flavor your food with it is constant. It is interesting that we discovered that drinking alcohol at lunch and dinner were positively related with HUA, but that drinking it during breakfast was negatively related with HUA. According to one study, increasing calorie intake in the morning may enhance metabolism and may be related to this [39]. Unfortunately, despite the fact that we discovered a link between drinking alcohol at breakfast and HUA, there has not been much research to support this, and more will be required in the future for scientific study.

### Strengths and Limitations

This study has several strengths. Firstly, it is the first and relatively large population study based on NHANES that reveals associations between dozens of food groups in daily life with HUA. Secondly, the associations reported in this study are relatively robust after adjustment for a variety of important confounding factors. Finally, not everyone is a nutritionist; thus, our study provides clues to help people choose certain foods at certain times to prevent HUA. We also recognize that this study has some limitations. Firstly, all dietary assessment methods inevitably introduce measurement error, although self-reported 24 h dietary recall is the most effective and commonly used dietary information capture tool in observational studies, it is nonetheless prone to measurement errors. For example, random errors may occur due to inaccurate recall. Secondly, we have the opportunity to control a range of potential confounding factors, but this study remains observational and other unknown confounding factors cannot be ruled out.

## 5. Conclusions

In this study, the consumption of fruits, milk and eggs, refined grains at breakfast and dinner, whole grains at lunch as well as reducing alcohol at lunch and dinner and *n*-3 fatty acid-rich seafood and poultry at dinner are negatively correlated with HUA.

## Figures and Tables

**Table 1:** Characteristics of participants with/without the HUA.

Characteristics	Total	Non-HUA	HUA	*p*-Value
	N = 41,230	n = 34,488	n = 6742	
Age (years)	43.53 ± 20.89	42.49 ± 20.61	48.67 ± 20.48	<0.001
Male, %	20,297 (49.23)	16,485 (46.83)	3812 (57.22)	<0.001
Non-Hispanic white, %	16,618 (40.31)	13,648 (39.77)	2970 (44.39)	<0.001
College graduate or above, %	7571 (18.36)	6345 (18.10)	1226 (18.79)	<0.001
Household income over $75,000, %	10,146 (24.61)	8672 (25.24)	1474 (20.72)	<0.001
Exercised regularly, %	11,920 (28.91)	10,320 (29.70)	1600 (23.95)	<0.001
Married, %	17,198 (41.71)	14,133 (40.68)	3065 (45.40)	<0.001
Smoking, %	6817 (16.53)	5739 (17.43)	1078 (16.01)	<0.001
Drinking, %	21,653 (52.52)	17,787 (51.66)	3866 (56.81)	<0.001
BMI, kg/m^2^	28.43 ± 7.09	27.62 ± 6.63	32.41 ± 7.88	<0.001
DM, %	9230 (22.39)	6799 (20.29)	2431 (35.93)	<0.001
Pre-DM, %	11,003 (26.69)	8638 (24.20)	2365 (34.11)	<0.001
Hypertension, %	14,129 (34.27)	10,267 (29.34)	3862 (53.63)	<0.001
Hyperlipidemia, %	26,658 (64.66)	21,256 (62.21)	5402 (80.68)	<0.001
CKD, %	6886 (16.7)	4792 (11.54)	2094 (26.02)	<0.001

Continuous variables are presented as mean ± standard deviation. Categorical variables are presented as numbers (%, percentage). BMI, body mass index. DM, diabetes mellitus. Pre-DM, prediabetes. CKD, Chronic Kidney Disease. HUA, Hyperuricemia.

**Table 2:** Characteristics of participants were divided according to whether or not they had HUA.

Characteristics	Total	Non-HUA	HUA	Adjust.*p*
	N = 41,230	n = 34,488	n = 6742	
f_whole (cup)	0.66 ± 1.08	0.68 ± 1.09	0.60 ± 1.06	0.002
f_citmlb (cup)	0.20 ± 0.64	0.20 ± 0.63	0.19 ± 0.68	0.355
f_juice (cup)	0.29 ± 0.80	0.30 ± 0.82	0.24 ± 0.69	<0.001
f_other (cup)	0.46 ± 0.82	0.47 ± 0.83	0.41 ± 0.77	0.002
f_total (cup)	0.95 ± 1.38	0.97 ± 1.40	0.84 ± 1.28	<0.001
v_drkgr (cup)	0.14 ± 0.37	0.14 ± 0.37	0.15 ± 0.38	0.883
v_redor_tomato (cup)	0.30 ± 0.41	0.30 ± 0.41	0.29 ± 0.40	0.813
v_redor_other (cup)	0.09 ± 0.23	0.09 ± 0.23	0.08 ± 0.22	0.182
v_redor_total (cup)	0.39 ± 0.47	0.39 ± 0.47	0.38 ± 0.46	0.429
v_starchy_potato (cup)	0.37 ± 0.60	0.36 ± 0.59	0.39 ± 0.61	0.049
v_starchy_other (cup)	0.08 ± 0.26	0.08 ± 0.26	0.08 ± 0.26	0.599
v_starchy_total (cup)	0.44 ± 0.66	0.44 ± 0.66	0.47 ± 0.67	0.049
v_other (cup)	0.55 ± 0.70	0.55 ± 0.71	0.54 ± 0.65	0.738
v_total (cup)	1.52 ± 1.25	1.52 ± 1.25	1.53 ± 1.21	0.599
v_legumes (cup)	0.11 ± 0.37	0.12 ± 0.37	0.11 ± 0.34	0.275
g_whole (oz)	0.83 ± 1.29	0.85 ± 1.29	0.76 ± 1.31	0.002
g_refined (oz)	5.86 ± 4.30	5.94 ± 4.40	5.46 ± 4.00	<0.001
g_total (oz)	6.69 ± 4.40	6.79 ± 4.50	6.22 ± 4.10	<0.001
d_milk (cup)	0.80 ± 1.08	0.83 ± 1.11	0.63 ± 0.89	<0.001
d_yogurt (cup)	0.06 ± 0.19	0.06 ± 0.19	0.05 ± 0.17	0.002
d_cheese (cup)	0.77 ± 1.03	0.78 ± 1.04	0.71 ± 0.94	0.002
d_total (cup)	1.66 ± 1.55	1.71 ± 1.58	1.41 ± 1.34	<0.001
pf_meat (oz)	1.65 ± 2.62	1.64 ± 2.61	1.71 ± 2.65	0.205
pf_curedmeat (oz)	1.02 ± 1.75	1.02 ± 1.74	1.01 ± 1.81	0.940
pf_organ (oz)	0.02 ± 0.31	0.02 ± 0.30	0.02 ± 0.40	0.738
pf_poult (oz)	1.53 ± 2.68	1.47 ± 2.59	1.84 ± 3.10	<0.001
pf_seafd_hi (oz)	0.15 ± 0.89	0.15 ± 0.84	0.20 ± 1.08	0.019
pf_seafd_low (oz)	0.44 ± 1.88	0.43 ± 1.82	0.49 ± 2.15	0.158
pf_mps_total (oz)	4.82 ± 4.20	4.72 ± 4.10	5.27 ± 4.60	<0.001
pf_eggs (oz)	0.53 ± 0.94	0.54 ± 0.96	0.49 ± 0.88	0.004
pf_soy (oz)	0.08 ± 0.37	0.08 ± 0.38	0.06 ± 0.30	0.002
pf_nutsds (oz)	0.71 ± 1.74	0.73 ± 1.75	0.62 ± 1.67	0.004
pf_legumes (oz)	0.46 ± 1.47	0.46 ± 1.49	0.42 ± 1.35	0.275
pf_total (oz)	6.14 ± 4.70	6.08 ± 4.70	6.44 ± 5.10	0.002
add_sugars (tsp)	18.20 ± 17.00	18.32 ± 17.00	17.59 ± 17.00	0.036
oils (grams)	25.67 ± 21.00	25.78 ± 22.00	25.17 ± 21.00	0.253
solid_fats (grams)	38.04 ± 28.00	38.36 ± 29.00	36.47 ± 27.00	0.006
a_drinks (nunber of drinks)	0.69 ± 1.85	0.63 ± 1.71	0.99 ± 2.41	<0.001

Continuous variables are presented as mean ± standard deviation. HUA, Hyperuricemia, oz, ounce, tsp, teaspoon.

## Data Availability

The datasets generated during and/or analyzed during the current study are available in the [NHANES] repository, [https://wwwn.cdc.gov/nchs/nhanes/default.aspx (accessed on 6 December 2022)].

## Supplementary Materials

The following supporting information can be downloaded at: https://www.mdpi.com/article/10.3390/nu15143109/s1, Table S1: Food Patterns Equivalents Database Components; Table S2: Characteristics of participants with/without the HUA in the breakfast cohort; Table S3: Characteristics of participants with/without the HUA in the lunch cohort; Table S4: Characteristics of participants with/without the HUA in the dinner cohort; Table S5: ORs and 95% CI for the association of variety of diets with and HUA stratified by gender; Table S6: ORs and 95% CI for the association of variety of diets with and HUA stratified by hypertension; Table S7: ORs and 95% CI for the association of variety of diets with and HUA stratified by CKD; Table S8: ORs and 95% CI for the association of variety of diets with and HUA stratified by drinking; Table S9: ORs and 95% CI for the association of variety of diets with HUA were excluded in populations with less than two years of follow-up.
