# Supplementary material for: The Association of Food Groups and Consumption Time with Hyperuricemia: The U.S. National Health and Nutrition Examination Survey, 2005–2018
Source: Nutrients. 2023 Jul 12;15(14):3109. doi: 10.3390/nu15143109 (PMC10386299; doi:10.3390/nu15143109)
Supplement: Supplementary file 1 [file nutrients-15-03109-s001.zip › nutrients-2461032-supplementary.pdf]

Supplementary Table S1. Food Patterns Equivalents Database Components

FPED component variable name	Foods
f_whole	Whole fruit
f_citmlb	Intact fruits (whole or cut) of citrus, melons, and berries
f_juice	Fruit juices, citrus and non-citrus
f_other	Intact fruits (whole or cut); excluding citrus, melons, and berries
f_total	Total intact fruits (whole or cut) and fruit juices
v_drkgr	Dark green vegetables
v_redor_tomato	Tomatoes and tomato products
v_redor_other	Other red and orange vegetables, excluding tomatoes and tomato products
v_redor_total	Total red and orange vegetables (tomatoes and tomato products + other red and orange vegetables)
v_starchy_potato	White potatoes
v_starchy_other	Other starchy vegetables, excluding white potatoes
v_starchy_total	Total starchy vegetables (white potatoes + other starchy vegetables)
v_other	Other vegetables not in the vegetable components listed above
v_total	Total dark green, red and orange, starchy, and other vegetables;

	excludes legumes
v_legumes	Beans and peas (legumes) computed as vegetables
g_whole	Grains defined as whole grains and contain the entire grain kernel — the bran, germ, and endosperm
g_refined	Refined grains that do not contain all of the components of the entire grain kernel
g_total	Total whole and refined grains
d_milk	Fluid milk, buttermilk, evaporated milk, dry milk, and calcium fortified soy milk
d_yogurt	Yogurt
d_cheese	Cheeses
d_total	Total milk, yogurt, cheese, and whey. For some foods, the total dairy values could be higher than the sum of D_MILK, D_YOGURT, and D_CHEESE because the Miscellaneous Dairy component composed of whey is not included in FPED as a separate variable.
pf_meat	Beef, veal, pork, lamb, and game meat; excludes organ meat and cured meat
pf_curedmeat	Frankfurters, sausages, corned beef, cured ham and luncheon meat that are made from beef, pork, or poultry
pf_organ	Organ meat from beef, veal, pork, lamb, game, and poultry
pf_poult	Chicken, turkey, Cornish hens, duck, goose, quail, and pheasant

	(game birds); excludes organ meat and cured meat
pf_seafd_hi	Seafood (finfish, shellfish, and other seafood) high in n-3 fatty acids
pf_seafd_low	Seafood (finfish, shellfish, and other seafood) low in n-3 fatty acids
pf_mps_total	Total of meat, poultry, seafood, organ meat, and cured meat
pf_eggs	Eggs (chicken, duck, goose, quail) and egg substitutes
pf_soy	Soy products, excluding calcium fortified soy milk (soymilk) and products made with raw (green) soybean
pf_nutsds	Peanuts, tree nuts, and seeds; excludes coconut
pf_legumes	Beans and peas (legumes) computed as protein foods
pf_total	Total meat, poultry, organ meat, cured meat, seafood, eggs, soy, and nuts and seeds; excludes legumes
add_sugars	Caloric sweeteners such as syrups and sugars and others defined as added sugars
oils	Fats naturally present in nuts, seeds, and seafood; all unhydrogentated vegetable oils, except palm oil, palm kernel oil, and coconut oils; the fat present in avocado and olives above the allowable amount; 50% of the fat present in stick and tub margarines and margarine spreads
solid_fats	Fats naturally present in meat, poultry, eggs, and dairy (lard, tallow, and butter); fully or partially hydrogenated oils;

	shortening; palm oil; palm kernel oil; coconut oils; fats naturally present in coconut meat and cocoa butter; and 50% of the fat present in stick and tub margarines and margarine spreads
a_drinks	Alcoholic beverages and alcohol (ethanol) added to foods after cooking

FPED, Food Patterns Equivalents Database

Supplementary Table S2. Characteristics of participants with/without the HUA in the breakfast cohort

Characteristics	Total N=29920	Non-HUA n=24962	HUA n=4958	*P*-value
Age (years)	45.37±21.33	44.21±21.12	51.1±19.99	< 0.001
Male, %	14507(48.49)	11796(45.88)	2711(55.21)	<0.001
Non-Hispanic white, %	14117(47.78)	11640(47.45)	2477(50.21)	<0.001
College graduate or above, %	6601(22.06)	5554(22.74)	1047(21.70)	<0.001
Household income over $75,000, %	8205(27.42)	6969(36.80)	1236(23.26)	<0.001
Exercised regularly, %	11920(27.91)	10320(29.70)	1600(21.95)	<0.001
Married, %	8214(44.45)	7131(42.60)	1083(48.72)	<0.001
Smoking, %	6817(16.53)	5739(18.43)	1078(14.01)	<0.001
Drinking, %	4709(55.74)	3992(53.16)	717(58.22)	<0.001
BMI, kg/m^2	28.33±7.08	27.54±6.64	32.29±7.73	<0.001
DM, %	6749(22.56)	4869(17.35)	1880(32.29)	<0.001
Pre-DM, %	8014(26.78)	6247(24.42)	1767(34.91)	<0.001
Hypertension, %	10958(36.62)	7930(31.11)	3028(57.12)	<0.001
Hyperlipidemia, %	19470(65.07)	15445(62.10)	4025(81.57)	<0.001
CKD, %	5321(17.78)	3663(11.92)	1658(27.79)	<0.001

Continuous variables are presented as mean±standard deviation. Categorical variables

are presented as numbers (%, percentage). BMI, body mass index. DM, diabetes mellitus, Pre-DM, prediabetes, CKD, Chronic Kidney Disease, HUA, hyperuricemia.

Supplementary Table S3. Characteristics of participants with/without the HUA in the lunch cohort

Characteristics	Total N=27927	Non-HUA n=23435	HUA n=4492	*P*-value
Age (years)	43.29±21.06	42.27±20.73	48.52±20.80	< 0.001
Male, %	13549(48.52)	11027(46.11)	2522(57.04)	<0.001
Non-Hispanic white, %	12779(45.76)	10564(44.89)	2215(49.33)	<0.001
College graduate or above, %	6153(22.03)	5194(20.99)	959(25.33)	<0.001
Household income over $75,000, %	7853(28.12)	6685(37.30)	1168(24.39)	<0.001
Exercised regularly, %	8010(28.68)	6957(30.24)	1053(21.26)	<0.001
Married, %	11643(41.69)	9562(40.93)	2081(46.05)	<0.001
Smoking, %	4193(15.01)	3561(16.55)	632(13.59)	<0.001
Drinking, %	14723(52.72)	12150(52.34)	2573(57.55)	<0.001
BMI, kg/m^2	28.25±7.16	27.47±6.74	32.22±7.80	<0.001
DM, %	5747(20.58)	4195(16.27)	1552(29.60)	<0.001
Pre-DM, %	7185(25.73)	5626(23.49)	1559(33.33)	<0.001
Hypertension, %	9158(32.79)	6658(28.71)	2500(52.77)	<0.001
Hyperlipidemia, %	17502(62.67)	13949(62.98)	3553(80.53)	<0.001
CKD, %	4551(16.3)	3215(11.40)	1336(25.79)	<0.001

Continuous variables are presented as mean±standard deviation. Categorical variables are presented as numbers (%, percentage). BMI, body mass index. DM, diabetes mellitus, Pre-DM, prediabetes, CKD, Chronic Kidney Disease, HUA, hyperuricemia.

Supplementary Table S4. Characteristics of participants with/without the HUA in the dinner cohort

Characteristics	Total N=27447	Non-HUA n=23082	HUA n=4365	*P*-value
Age (years)	41.56±20.61	40.66±20.25	46.3±20.65	< 0.001
Male, %	13380(48.75)	10866(46.59)	2514(59.10)	<0.001
Non-Hispanic white, %	11324(41.26)	9456(41.15)	1868(43.18)	<0.001
College graduate or above, %	6067(22.1)	5123(22.29)	944(21.11)	<0.001
Household income over $75,000, %	7764(28.29)	6610(29.53)	1154(24.35)	<0.001
Exercised regularly, %	8339(30.38)	7224(31.10)	1115(26.52)	<0.001
Married, %	10822(39.43)	8893(36.24)	1929(44.80)	<0.001
Smoking, %	4313(15.71)	3623(16.34)	690(15.58)	<0.001
Drinking, %	14751(53.74)	12161(52.66)	2590(59.25)	<0.001
BMI, kg/m^2	28.09±7.22	27.29±6.74	32.29±8.00	<0.001
DM, %	5368(19.56)	3887(15.11)	1481(28.51)	<0.001
Pre-DM, %	6959(25.35)	5443(23.13)	1516(33.44)	<0.001
Hypertension, %	8517(31.03)	6165(26.68)	2352(50.57)	<0.001
Hyperlipidemia, %	16852(61.4)	13430(57.62)	3422(79.39)	<0.001
CKD, %	4138(15.08)	2935(10.36)	1203(22.84)	<0.001

Continuous variables are presented as mean±standard deviation. Categorical variables

are presented as numbers (%, percentage). BMI, body mass index. DM, diabetes mellitus, Pre-DM, prediabetes, CKD, Chronic Kidney Disease, HUA, hyperuricemia.

Supplementary Table S5. ORs and 95%CI for the association of variety of diets with and HUA stratified by gender

Characteristics	Male (n = 20297)	Female (n = 20933)
	OR (95%CI)	OR (95%CI)
f_whole	0.960(0.913,1.009)	0.926(0.857, 1.001)
f_citmlb	1.002(0.920,1.092)	0.926(0.782, 1.097)
f_juice	0.939(0.872,1.011)	0.803(0.694, 0.930)
f_other	0.930(0.868,0.995)	0.920(0.849, 0.998)
f_total	0.955(0.915,0.996)	0.897(0.837, 0.962)
v_drkgr	0.977(0.837,1.141)	1.235(1.053, 1.448)
v_redor_tomato	0.949(0.835,1.078)	0.931(0.764, 1.134)
v_redor_other	0.740(0.565,0.971)	1.088(0.890, 1.329)
v_redor_total	0.902(0.800,1.017)	0.985(0.848, 1.146)
v_starchy_potato	1.054(0.969,1.147)	0.896(0.779, 1.030)
v_starchy_other	0.919(0.774,1.092)	1.203(0.924, 1.565)
v_starchy_total	1.035(0.959,1.117)	0.943(0.839, 1.060)
v_other	0.945(0.862,1.035)	1.002(0.923, 1.089)
v_total	0.976(0.928,1.027)	1.015(0.967, 1.066)
v_legumes	0.916(0.788,1.064)	0.934(0.715, 1.220)
g_whole	0.950(0.910,0.991)	0.943(0.884, 1.007)
g_refined	0.960(0.947,0.972)	0.962(0.942, 0.982)
g_total	0.957(0.944,0.969)	0.957(0.937, 0.978)

d_milk	0.824(0.776,0.875)	0.835(0.767, 0.909)
d_yogurt	0.731(0.501,1.067)	0.736(0.519, 1.045)
d_cheese	0.909(0.860,0.960)	0.921(0.850, 0.998)
d_total	0.868(0.828,0.909)	0.866(0.818, 0.916)
pf_meat	0.984(0.967,1.002)	0.991(0.954, 1.030)
pf_curedmeat	0.961(0.933,0.990)	0.951(0.903, 1.002)
pf_organ	0.981(0.873,1.103)	1.061(0.890, 1.266)
pf_poult	1.041(1.026,1.057)	1.062(1.034, 1.091)
pf_seafd_hi	1.038(0.993,1.085)	1.121(1.051, 1.194)
pf_seafd_low	1.012(0.990,1.034)	0.983(0.942, 1.027)
pf_mps_total	1.008(0.996,1.020)	1.022(1.001, 1.043)
pf_eggs	0.907(0.857,0.960)	0.845(0.771, 0.926)
pf_soy	0.873(0.750,1.015)	1.051(0.872, 1.265)
pf_nutsds	0.980(0.951,1.010)	0.955(0.907, 1.006)
pf_legumes	0.979(0.942,1.016)	0.982(0.919, 1.050)
pf_total	0.999(0.988,1.011)	1.002(0.985, 1.020)
add_sugars	0.997(0.993,1.000)	0.996(0.990, 1.001)
oils	0.998(0.995,1.001)	0.996(0.992, 0.999)
solid_fats	0.994(0.992,0.996)	0.995(0.992, 0.998)
a_drinks	1.084(1.060,1.108)	1.152(1.097, 1.211)

Results were adjusted for age, race, marriage, education, smoking, drinking, income, exercise, BMI, DM, pre-DM, hyperlipidemia, hypertension and CKD. DM, diabetes

mellitus, Pre-DM, prediabetes, CKD, Chronic Kidney Disease, HUA, hyperuricemia.

Supplementary Table S6. ORs and 95%CI for the association of variety of diets with and HUA stratified by hypertension

Characteristics	Non-hypertension (n = 27101)	Hypertension (n = 14129)
	OR (95%CI)	OR (95%CI)
f_whole	0.968(0.907,1.034)	0.934(0.877,0.994)
f_citmlb	0.984(0.882,1.098)	0.964(0.866,1.072)
f_juice	0.921(0.850,0.998)	0.889(0.806,0.981)
f_other	0.955(0.885,1.031)	0.910(0.839,0.988)
f_total	0.955(0.910,1.002)	0.924(0.874,0.977)
v_drkgr	1.125(0.967,1.308)	1.044(0.918,1.187)
v_redor_tomato	1.071(0.918,1.249)	0.863(0.762,0.978)
v_redor_other	0.890(0.693,1.144)	0.861(0.646,1.146)
v_redor_total	1.024(0.897,1.167)	0.863(0.766,0.973)
v_starchy_potato	0.979(0.895,1.071)	1.037(0.935,1.150)
v_starchy_other	1.165(0.948,1.433)	0.832(0.633,1.093)
v_starchy_total	1.024(0.897,1.167)	1.008(0.918,1.106)
v_other	0.984(0.917,1.056)	0.965(0.866,1.076)
v_total	1.012(0.969,1.056)	0.974(0.921,1.031)
v_legumes	0.978(0.818,1.168)	0.903(0.747,1.090)
g_whole	0.931(0.881,0.983)	0.962(0.925,1.001)
g_refined	0.961(0.946,0.976)	0.968(0.952,0.984)

g_total	0.956(0.941,0.971)	0.965(0.949,0.981)
d_milk	0.812(0.764,0.864)	0.843(0.774,0.918)
d_yogurt	0.765(0.527,1.110)	0.754(0.547,1.039)
d_cheese	0.904(0.854,0.957)	0.946(0.883,1.013)
d_total	0.854(0.816,0.894)	0.893(0.841,0.948)
pf_meat	0.997(0.975,1.020)	0.985(0.957,1.013)
pf_curedmeat	0.942(0.907,0.979)	0.977(0.947,1.009)
pf_organ	1.005(0.870,1.160)	0.977(0.883,1.081)
pf_poult	1.049(1.030,1.069)	1.049(1.026,1.073)
pf_seafd_hi	1.053(0.999,1.109)	1.090(1.014,1.171)
pf_seafd_low	1.026(0.999,1.054)	0.981(0.952,1.010)
pf_mps_total	1.020(1.007,1.033)	1.009(0.993,1.024)
pf_eggs	0.883(0.825,0.945)	0.903(0.847,0.963)
pf_soy	0.925(0.783,1.092)	0.990(0.806,1.216)
pf_nutsds	0.983(0.950,1.019)	0.963(0.928,0.999)
pf_legumes	0.994(0.951,1.039)	0.975(0.930,1.022)
pf_total	1.008(0.995,1.022)	0.997(0.983,1.010)
add_sugars	0.998(0.994,1.002)	0.995(0.991,1.000)
oils	0.998(0.995,1.001)	0.997(0.994,1.001)
solid_fats	0.994(0.992,0.997)	0.996(0.993,0.998)
a_drinks	1.113(1.086,1.142)	1.090(1.052,1.130)

Results were adjusted for age, gender, race, marriage, education, smoking, drinking,

income, exercise, BMI, DM, pre-DM, hyperlipidemia and CKD. DM, diabetes mellitus, Pre-DM, prediabetes, CKD, Chronic Kidney Disease, HUA, hyperuricemia.

Supplementary Table S7. ORs and 95%CI for the association of variety of diets with and HUA stratified by CKD

Characteristics	Non-CKD (n = 34344)	CKD (n = 6886)
	OR (95%CI)	OR (95%CI)
f_whole	0.943(0.895,0.995)	0.985(0.894,1.085)
f_citmlb	0.950(0.864,1.046)	1.055(0.906,1.227)
f_juice	0.901(0.830,0.979)	0.901(0.799,1.015)
f_other	0.934(0.878,0.993)	0.929(0.821,1.050)
f_total	0.933(0.892,0.976)	0.962(0.894,1.036)
v_drkgr	1.095(0.970,1.237)	1.121(0.930,1.351)
v_redor_tomato	0.976(0.854,1.114)	0.920(0.749,1.129)
v_redor_other	0.832(0.671,1.032)	1.164(0.857,1.583)
v_redor_total	0.942(0.840,1.057)	0.982(0.815,1.182)
v_starchy_potato	1.017(0.938,1.102)	0.943(0.811,1.096)
v_starchy_other	0.998(0.825,1.207)	1.005(0.668,1.512)
v_starchy_total	1.014(0.943,1.090)	0.954(0.836,1.088)
v_other	0.980(0.916,1.048)	0.992(0.864,1.139)
v_total	0.998(0.958,1.041)	0.992(0.917,1.073)
v_legumes	0.956(0.833,1.098)	0.879(0.654,1.181)
g_whole	0.952(0.915,0.990)	0.924(0.870,0.982)
g_refined	0.961(0.949,0.973)	0.973(0.948,0.999)
g_total	0.958(0.946,0.970)	0.965(0.941,0.990)

d_milk	0.828(0.782,0.877)	0.831(0.756,0.914)
d_yogurt	0.845(0.630,1.134)	0.575(0.348,0.950)
d_cheese	0.898(0.850,0.949)	1.063(0.965,1.171)
d_total	0.864(0.827,0.903)	0.917(0.862,0.976)
pf_meat	0.987(0.968,1.006)	1.002(0.967,1.037)
pf_curedmeat	0.952(0.921,0.983)	0.980(0.936,1.025)
pf_organ	0.989(0.893,1.097)	0.970(0.775,1.214)
pf_poult	1.057(1.043,1.071)	1.019(0.985,1.054)
pf_seafd_hi	1.062(1.014,1.113)	1.116(1.030,1.209)
pf_seafd_low	1.007(0.986,1.029)	0.999(0.955,1.046)
pf_mps_total	1.015(1.005,1.026)	1.012(0.989,1.035)
pf_eggs	0.889(0.843,0.937)	0.892(0.810,0.982)
pf_soy	0.967(0.855,1.093)	0.870(0.690,1.096)
pf_nutsds	0.970(0.942,0.999)	1.000(0.948,1.054)
pf_legumes	0.989(0.955,1.023)	0.969(0.900,1.043)
pf_total	1.003(0.992,1.014)	1.003(0.984,1.023)
add_sugars	0.997(0.994,1.000)	0.995(0.989,1.002)
oils	0.998(0.995,1.000)	0.997(0.993,1.002)
solid_fats	0.994(0.992,0.996)	0.999(0.995,1.002)
a_drinks	1.101(1.075,1.127)	1.086(1.031,1.145)

Results were adjusted for age, gender, race, marriage, education, smoking, drinking, income, exercise, BMI, DM, pre-DM, hyperlipidemia and hypertension. DM, diabetes

mellitus, Pre-DM, prediabetes, CKD, Chronic Kidney Disease, HUA, hyperuricemia.

Supplementary Table S8. ORs and 95%CI for the association of variety of diets with and HUA stratified by drinking

Characteristics	Non-drinking (n = 19577)	Drinking (n = 21653)
	OR (95%CI)	OR (95%CI)
f_whole	0.943(0.873,1.018)	0.960(0.902, 1.022)
f_citmlb	0.982(0.869,1.110)	0.998(0.993, 1.003)
f_juice	0.892(0.791,1.006)	0.912(0.839, 0.992)
f_other	0.915(0.829,1.011)	0.943(0.872, 1.021)
f_total	0.931(0.869,0.999)	0.947(0.899, 0.998)
v_drkgr	1.174(0.947,1.455)	1.074(0.940, 1.227)
v_redor_tomato	1.113(0.918,1.349)	0.932(0.811, 1.071)
v_redor_other	0.655(0.432,0.994)	0.888(0.701, 1.125)
v_redor_total	0.976(0.820,1.161)	0.922(0.815, 1.041)
v_starchy_potato	1.053(0.905,1.225)	0.998(0.910, 1.094)
v_starchy_other	0.946(0.723,1.236)	1.028(0.833, 1.268)
v_starchy_total	1.035(0.902,1.187)	1.002(0.927, 1.084)
v_other	1.080(0.955,1.222)	0.960(0.885, 1.041)
v_total	1.047(0.967,1.135)	0.983(0.937, 1.032)
v_legumes	0.844(0.674,1.058)	0.932(0.777, 1.117)
g_whole	0.984(0.931,1.040)	0.945(0.905, 0.988)
g_refined	0.978(0.958,0.998)	0.959(0.946, 0.972)
g_total	0.977(0.957,0.997)	0.955(0.942, 0.968)

d_milk	0.799(0.737,0.867)	0.820(0.765, 0.878)
d_yogurt	0.617(0.424,0.898)	0.896(0.651, 1.233)
d_cheese	0.939(0.868,1.017)	0.912(0.856, 0.970)
d_total	0.849(0.804,0.896)	0.872(0.828, 0.918)
pf_meat	0.996(0.959,1.03)	0.989(0.969, 1.009)
pf_curedmeat	0.956(0.901,1.015)	0.966(0.937, 0.995)
pf_organ	1.050(0.889,1.240)	0.969(0.850, 1.104)
pf_poult	1.071(1.038,1.106)	1.047(1.032, 1.064)
pf_seafd_hi	1.175(1.066,1.295)	1.061(1.012, 1.111)
pf_seafd_low	0.963(0.919,1.010)	1.015(0.992, 1.038)
pf_mps_total	1.024(1.003,1.046)	1.015(1.003, 1.027)
pf_eggs	0.876(0.797,0.964)	0.891(0.837, 0.948)
pf_soy	1.004(0.786,1.284)	0.951(0.828, 1.093)
pf_nutsds	0.996(0.939,1.057)	0.967(0.936, 0.999)
pf_legumes	0.959(0.907,1.014)	0.982(0.939, 1.028)
pf_total	1.012(0.993,1.032)	1.001(0.989, 1.014)
add_sugars	0.996(0.990,1.002)	0.997(0.994, 1.000)
oils	1.002(0.998,1.007)	0.997(0.994, 1.000)
solid_fats	0.994(0.990,0.997)	0.995(0.992, 0.997)
a_drinks	1.142(0.863,1.511)	1.093(1.070, 1.117)

Results were adjusted for age, gender, race, marriage, education, smoking, income, exercise, BMI, DM, pre-DM, hyperlipidemia, hypertension and CKD. DM, diabetes

mellitus, Pre-DM, prediabetes, CKD, Chronic Kidney Disease, HUA, hyperuricemia.

Supplementary Table S9. ORs and 95%CI for the association of variety of diets with HUA were excluded in populations with less than two years of follow-up

Characteristics	OR (95%CI)
f_whole	0.950(0.904,0.998)
f_citmlb	0.984(0.904,1.070)
f_juice	0.907(0.846,0.973)
f_other	0.927(0.875,0.981)
f_total	0.939(0.900,0.978)
v_drkgr	1.049(0.944,1.166)
v_redor_tomato	0.950(0.849,1.063)
v_redor_other	0.878(0.718,1.073)
v_redor_total	0.932(0.844,1.030)
v_starchy_potato	1.000(0.924,1.082)
v_starchy_other	0.999(0.841,1.187)
v_starchy_total	1.000(0.934,1.070)
v_other	0.955(0.891,1.024)
v_total	0.980(0.943,1.018)
v_legumes	0.917(0.796,1.055)
g_whole	0.952(0.921,0.985)
g_refined	0.963(0.951,0.974)
g_total	0.959(0.948,0.971)
d_milk	0.837(0.798,0.879)

d_yogurt	0.752(0.565,1.001)
d_cheese	0.917(0.874,0.962)
d_total	0.875(0.842,0.909)
pf_meat	0.988(0.970,1.006)
pf_curedmeat	0.951(0.925,0.978)
pf_organ	1.009(0.912,1.117)
pf_poult	1.054(1.040,1.068)
pf_seafd_hi	1.066(1.022,1.112)
pf_seafd_low	1.008(0.987,1.030)
pf_mps_total	1.015(1.005,1.026)
pf_eggs	0.884(0.838,0.932)
pf_soy	0.940(0.831,1.063)
pf_nutsds	0.974(0.948,1.000)
pf_legumes	0.979(0.946,1.013)
pf_total	1.003(0.992,1.014)
add_sugars	0.998(0.995,1.001)
oils	0.998(0.996,1.000)
solid_fats	0.994(0.992,0.996)
a_drinks	1.103(1.079,1.128)

Results were adjusted for age, gender, race, marriage, education, smoking, drinking, income, exercise, BMI, DM, pre-DM, hyperlipidemia, hypertension and CKD. DM, diabetes mellitus, Pre-DM, prediabetes, CKD, Chronic Kidney Disease, HUA, hyperuricemia.
